# Supplementary material for: Newly Designed Mesoporous Silica and Organosilica Nanostructures Based on Pentablock Copolymer Templates in Weakly Acidic Media
Source: Nanomaterials (Basel). 2021 Sep 27;11(10):2522. doi: 10.3390/nano11102522 (PMC8538493; doi:10.3390/nano11102522)
Supplement: Supplementary file 1 [file nanomaterials-11-02522-s001.zip › nanomaterials-1352932-supplementary.pdf]

Supporting Information

Newly Designed Mesoporous Silica and Organosilica Nanostructures Based on Pentablock Copolymer Templates in Weakly Acidic Media

Nabanita Pal [1], Young Sunwoo [2], Jae-Seo Park [2], Taeyeon Kim [2] and Eun-Bum Cho [2,*]

[1] Department of Physics and Chemistry, Mahatma Gandhi Institute of Technology, Gandipet, Hyderabad 500075, India; nabanitapal_chem@mgit.ac.in

[2] Department of Fine Chemistry, Seoul National University of Science and Technology, Seoul 01811, Korea; youngsw02@gmail.com (Y.S.); dedswe2@gmail.com (J.-S.P.); ktyeon91@gmail.com (T.K.)

* Correspondence: echo@seoultech.ac.kr

**Figure S1**	SEM images of mesoporous silica samples: PMSB-2 (a-1, a-2), PMSB-3 (b-1, b-2), PMSB-4 (c-1, c-2).
**Figure S2**	SEM images of mesoporous organosilica samples: PMOA-1 (a-1, a-2), PMOA-2 (b-1, b-2), PMOA-3 (c-1, c-2), PMOA-4 (d-1, d-2), PMOB-1 (e-1, e-2), PMOB-2 (f-1, f-2), PMOB-3 (g-1, g-2), PMOB-4 (h-1, h-2).
**Figure S3**	SEM images of mesoporous organosilica samples: PMOBH-1 (a-1, a-2), PMOBH-2 (b-1, b-2), PMOBH-3 (c-1, c-2), PMOBH-4 (d-1, d-2).

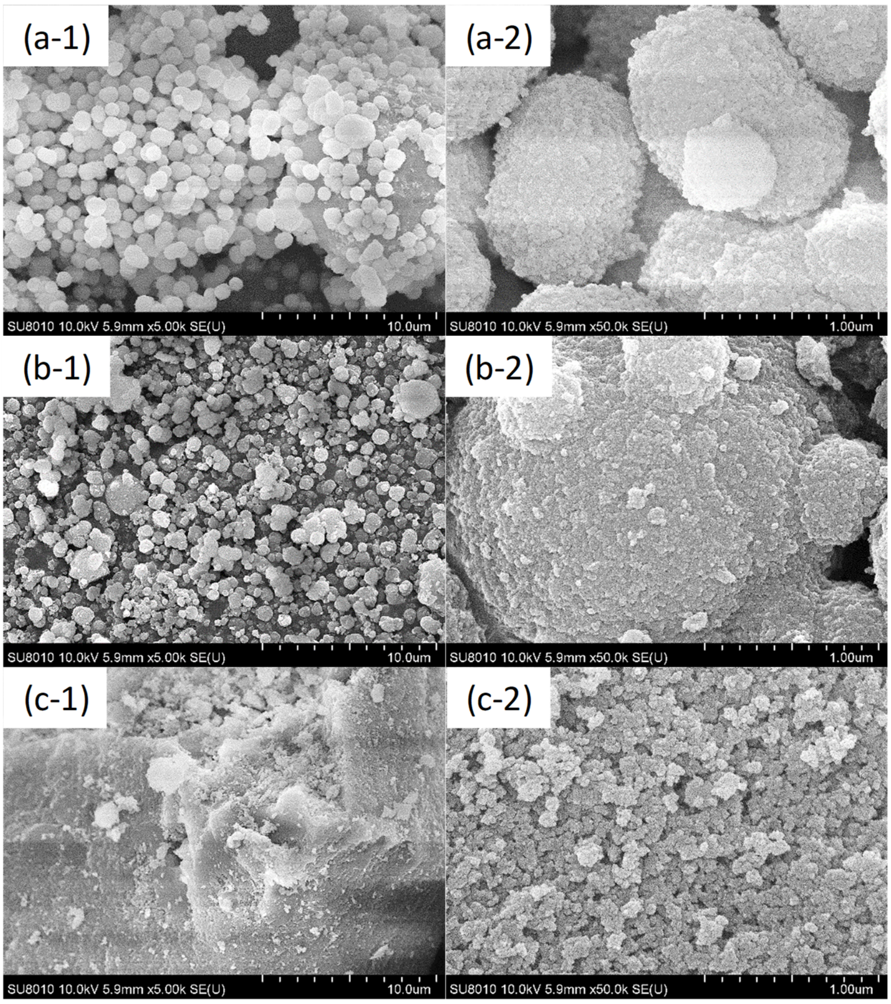

Figure S1. SEM images of mesoporous silica samples: PMSB-2 (**a-1**, **a-2**), PMSB-3 (**b-1**, **b-2**), PMSB-4 (**c-1**, **c-2**).

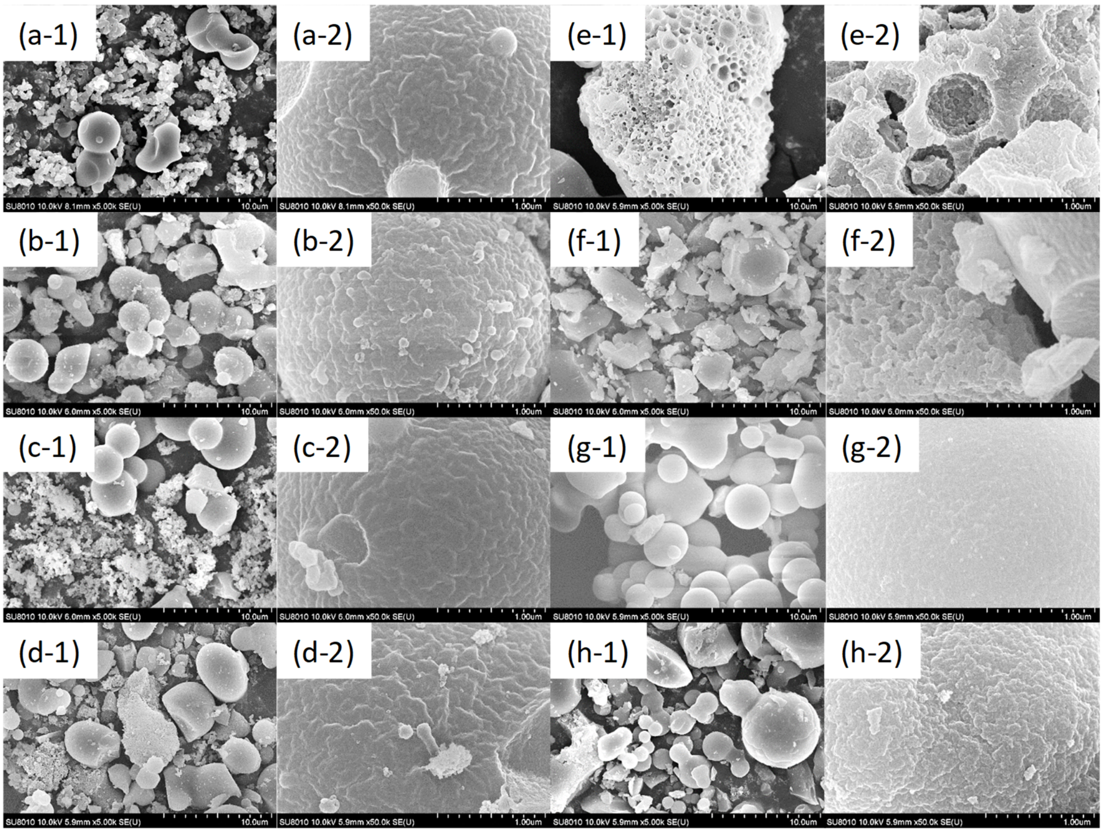

Figure S2. SEM images of mesoporous organosilica samples: PMOA-1 (**a-1**, **a-2**), PMOA-2 (**b-1**, **b-2**), PMOA-3 (**c-1**, **c-2**), PMOA-4 (**d-1**, **d-2**), PMOB-1 (**e-1**, **e-2**), PMOB-2 (**f-1**, **f-2**), PMOB-3 (**g-1**, **g-2**), PMOB-4 (**h-1**, **h-2**).

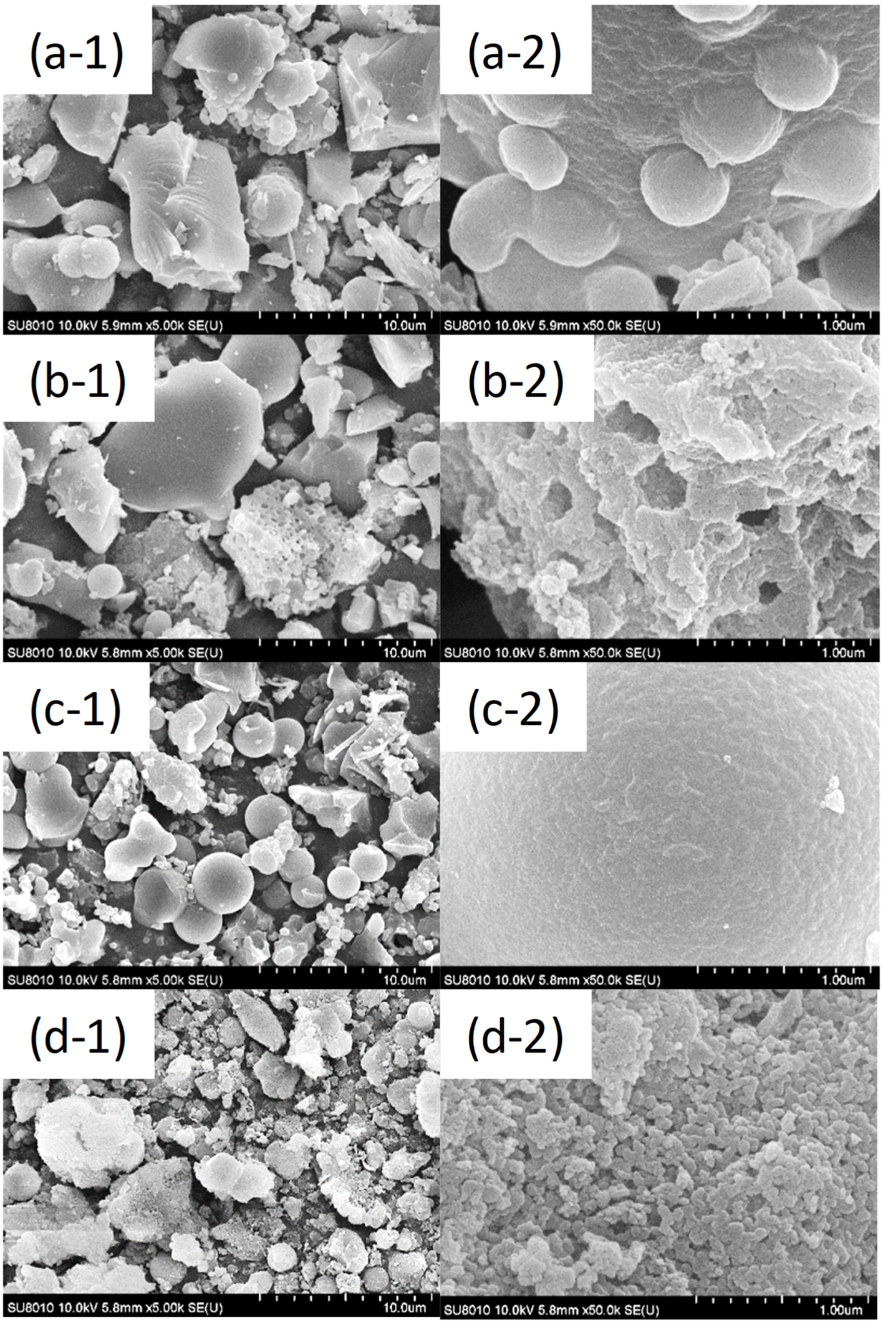

Figure S3. SEM images of mesoporous organosilica samples: PMOBH-1 (**a-1**, **a-2**), PMOBH-2 (**b-1**, **b-2**), PMOBH-3 (**c-1**, **c-2**), PMOBH-4 (**d-1**, **d-2**).
